# Comparative Transcriptomics Analysis of Foot-and-Mouth Disease Virus-Infected Cell Model Systems

**DOI:** 10.3390/vetsci12020107

**Published:** 2025-02-01

**Authors:** Haibin Ma, Zhenzhen Zheng, Min Liu, Mengsi Zhang, Xiaoyun Qu, Jingqiang Ren, Ming Liao

**Affiliations:** 1 Guangdong Provincial Key Laboratory of Zoonosis Prevention and Control, College of Veterinary Medicine, South China Agricutural University, Guangzhou 510642, China; mahaibin9111@stu.scau.edu.cn (H.M.); xyqu@scau.edu.cn (X.Q.); 2 Wenzhou Key Laboratory for Virology and Immunology, Institute of Virology, Wenzhou University, Wenzhou 325035, China; 23451039051@stu.wzu.edu.cn (Z.Z.); 22451335023@stu.wzu.edu.cn (M.L.); 23451040023@stu.wzu.edu.cn (M.Z.)

**Keywords:** foot-and-mouth disease virus (FMDV), transcriptomics, differentially expressed gene (DEG), enrichment analysis

## Abstract

The highly contagious FMDV is the agent responsible for foot-and-mouth disease, significantly impacting animals with cloven hooves and incurring substantial economic losses globally. The FMDV genome, composed of single-stranded RNA, consists of approximately 8500 nucleotides and harbors a single open reading frame (ORF) encoding both structural and non-structural proteins vital for the virus’s pathogenicity and replication. BHK-21 (baby hamster kidney) cells are the optimal cell line for FMDV culture due to their robust viral replication ability and high infection susceptibility. The insufficient elucidation of the host response to FMDV hampers progress towards the establishment of precise therapeutic interventions. To fill this void in understanding, samples from FMDV-challenged and control BHK-21 cells were systematically procured, with comprehensive transcriptome sequencing subsequently undertaken to delineate the gene expression landscapes of each group. A total of 4018 differentially expressed genes were identified, of which 2044 were downregulated and 1974 were upregulated. The data indicate that FMDV infection significantly enhances transcription initiation in BHK-21. According to GO and KEGG enrichment analysis, FMDV affects a number of immune-related processes as well as the movement of chemicals within cells. In the analysis of the protein–protein interaction network, Fos, Flt3lg, Rpl22l1, Ifi35, Ep300, and Rps16 emerged as pivotal hub proteins, underscoring their significant roles within the cellular interactome. The RT-qPCR experiment of Lgfb5, Ler2, Vgll3, and Ahr verified that the DEGs’ expression profiles matched the results of the RNA-seq investigation. The study’s findings have enhanced our understanding of the molecular pathways underlying FMDV pathogenesis and host interactions. Furthermore, the identification of key genes could serve as potential targets for therapeutic strategies and diagnostic tools, thereby enhancing control measures for livestock foot-and-mouth disease and mitigating its economic impact.

## Simple Summary

The foot-and-mouth disease virus (FMDV) is a highly contagious pathogen that severely affects cloven-hoofed animals, leading to significant economic losses worldwide. Our limited understanding of how hosts respond to FMDV has hindered the development of effective treatments. To address this, we sampled and sequenced the transcriptomes of FMDV-infected and control BHK-21 cells. This study encompasses comprehensive analyses of differential gene expression, functional enrichments, genetic variations, alternative splicing, protein interaction networks, and gene set enrichments. The study found that following foot-and-mouth disease virus infection, a total of 4018 differentially expressed genes were identified in the cells, involving significant changes in immune-related pathways and key hub proteins, with RT-qPCR confirming the accuracy of the RNA-seq results. These data indicate that the virus infection significantly enhances transcription initiation within the cells and highlights the important roles of several key proteins in the cellular interactome. To our knowledge, this is the first transcriptomic analysis of BHK-21 cells and blank control cells after FMDV infection. This research enhances our knowledge of FMDV’s effects on host cells and identifies potential targets for new treatments and diagnostics, which could help control the disease and reduce its economic impact.

## 1. Introduction

The foot-and-mouth disease virus, a member of the Aphthovirus genus in the Picornaviridae family, is the cause of foot-and-mouth disease (FMD) [1]. This highly contagious disease impacts a wide range of cloven-hoofed animals, such as pigs, sheep, goats, cattle, camelids, and deer, causing substantial economic damage in the agricultural sector worldwide [2]. The FMDV genome, approximately 8.5 kb in length, encodes a single polyprotein. This polyprotein is cleaved by the leader protease (Lpro), 2A, and 3C proteases (3Cpro) into eight nonstructural and three structural proteins (VP0, VP1, and VP3) [3]. The assembly of the FMDV capsid begins with the formation of the protomer by VP0, VP1, and VP3, which then come together to form pentamers and a 75S empty capsid. After maturation cleavage of VP0 into VP2 and VP4 and encapsulation of the progeny RNA, the infectious 146S virion is produced [4].

RNA sequencing, or RNA-seq, harnesses next-generation sequencing technology to quantify RNA molecules within a biological sample, providing a comprehensive transcriptome that reflects the gene expression profile of an organism. This transcriptome includes all mRNA molecules and offers insights into the functional state of cells [5]. RNA-seq is particularly powerful for assessing transcriptomic changes, even at the single-cell level within specific populations, which is crucial for understanding the complex interactions between FMDV and its host. Comparative transcriptomics enables a detailed analysis of gene expression dynamics, alternative splicing, post-transcriptional modifications, gene fusion events, and single nucleotide polymorphisms (SNPs) between different conditions, such as infected versus non-infected cells. In the context of FMDV, this approach is instrumental in elucidating how the virus manipulates the host transcriptome, contributing to pathogenesis. While big DNA viruses like human cytomegalovirus and African swine fever virus possess large genomes and intricate transcriptomes in infected cells, RNA viruses such as picornaviruses, including FMDV, have simpler transcriptomes characterized by a single RNA genome [6]. However, the simplicity of the picornavirus transcriptome does not diminish its significance; rather, it underscores the need to investigate the virus–host interaction at a deeper level.

Through both virus-specific and non-specific methods, FMDV causes a variety of metabolic and biochemical changes in cells [7]. The 2B protein, or viroporin, has been shown to mediate FMDV replication by activating the NLRP3 inflammasome, a member of the Nod-like receptor family [2]. Additionally, by preventing RIG-I’s protein production, the 2B protein negates its antiviral activities. Additionally, FMDV infection affects host transcription levels [8]. According to one earlier study, the FMDV 3A protein helps to prevent harmful effects. For instance, decreased phosphorylation of IRF3 is the consequence of the FMDV 3A protein’s interaction with DEAD-box helicase 56 [9]. The FMDV 3A protein also interacts with annexin-A1 to inhibit the production of type I interferon (IFN-I), thereby enhancing FMDV replication [9]. Evidence indicates that FMDV infection significantly alters the cellular proteome and metabolome. Changes in the cellular transcriptome are closely linked to viral-induced modifications in the cellular proteome. By analyzing the transcriptomic data, we seek to identify key host factors and pathways that may be targeted to modulate the virus’s life cycle, potentially leading to the development of novel antiviral strategies.

BHK-21 cells are extensively utilized for the investigation and manufacturing of FMDV due to their high susceptibility to this virus. These cells facilitate the swift and efficient replication of FMDV, which is vital for both research endeavors and vaccine production. The well-documented nature of BHK-21 cells means that their reaction to viral infections is thoroughly understood, positioning them as a dependable model for transcriptomic sequencing research. The standardized culture and manipulation of BHK-21 cells ensure experimental uniformity, which is indispensable for data comparison and result replication. Moreover, the distinct cytopathic effect (CPE) induced by FMDV in BHK-21 cells simplifies the monitoring of infection progression [1]. This attribute, coupled with their significance in the production of inactivated FMD vaccines, makes BHK-21 cells the optimal choice for exploring the virus–host cell interaction and for devising effective strategies against FMDV. Consequently, BHK-21 cells were employed as the model system in this study to examine the host response to FMDV infection [3].

## 2. Methods

### 2.1. Virus and Cell Line

Dulbecco’s modified Eagle’s medium (DMEM) from Shenggong, Shanghai, China, was used to cultivate the BHK-21 cell line, which was generated from baby hamster kidney cells at 37 °C in 5% CO_2_. A total of 100 U/mL of penicillin, 100 μg/mL of streptomycin, and 10% fetal bovine serum (Gibco, CA, USA) were added to the culture medium. The Changchun Vet Research Laboratory of the Chinese Academy of Agricultural Sciences, located in Changchun, China, is responsible for maintaining the serotype O of FMDV. The virus was propagated in BHK-21 cells, and a 50% tissue culture infective dose (TCID50) assay was employed to quantify the viral titer within these cells.

### 2.2. Preparing the Sample

Six-well cell culture plates were seeded with BHK-21 cells, which were then cultivated at 37 °C. Three wells were selected for FMDV incubation at an MOI of 0.1 once the cells reached 90% confluence. The remaining wells served as untreated controls to ensure non-contamination. Consequently, three samples (S1, S2, and S3) were contaminated, whereas three samples (C1, C2, and C3) were not. The supernatant from all six wells was meticulously collected at 12 h post-inoculation. Following three delicate washes with PBS, total RNA extraction was carried out on the cell monolayers using TRIzol reagent (Shenggong, Shanghai, China) in accordance with the manufacturer’s protocol. For the extraction of RNA from BHK-21 cells using the Sangon Biotech RNA extraction kit, the procedure is as follows: begin by harvesting the cells and resuspending them in lysis buffer, then add an RNAase inhibitor to safeguard against RNA degradation. Proceed with a phenol–chloroform extraction to differentiate RNA from proteins and DNA, followed by RNA precipitation with isopropanol and purification through a 70% ethanol wash to eliminate contaminants. Conclude by dissolving the purified RNA in RNase-free water and evaluating its concentration and purity with a spectrophotometer. The NanoDrop spectrophotometer, provided by Thermo Fisher (Waltham, MA, USA), was utilized to assess the concentration, quality, and integrity of the total RNA. Five micrograms of RNA was extracted from each group to prepare the RNA samples.

### 2.3. Analysis of RNA-Seq

Sequencing library preparation was modified from standard procedures [10]. mRNA was purified using Oligo(dT) magnetic beads to remove ribosomal RNA from the total RNA sample. This mRNA was then fragmented and used as templates to generate cDNAs. The first strand of cDNA was created using reverse transcriptase and random hexamer primers. The second strand was made using the original cDNA strand, buffer solution, dNTPs, DNA polymerase I, and RNase H. Any remaining overhangs were converted to blunt ends through polymerase and exonuclease reactions. To facilitate hybridization, Illumina paired-end adapter oligonucleotides were ligated to the 3′ ends of DNA fragments after adenylation. The AMPure XP system (Beckman Coulter, Beverly, MA, USA) was used to size-select cDNA fragments between 250 and 300 base pairs. The Illumina PCR Primer Cocktail was used to enrich DNA fragments with adaptor molecules ligated at both ends in a 15-cycle PCR reaction. After being purified by the AMPure XP system, the products were measured using the Agilent high-sensitivity DNA test on the Agilent 2100 Bioanalyzer. Lastly, the Illumina NovaSeq 6000 platform was used to sequence the sequencing libraries in order to produce picture files.

### 2.4. Control of Quality and Mapping of Reads

The sequencing platform’s software processed the image files, generating original data in FASTQ format or raw data. These data contained numerous adapters and low-quality reads. To enhance data quality, the sequencing data were filtered using the Cutadapt (v1.15) program. This produced clean data, or high-quality sequences, that could be used for additional analysis. The golden hamster’s genome (Mesocricetus auratus, Genbank No.: gcf_01763985_1) served as the reference genome. Subsequently, HISAT2 (v2.0.5) was employed to individually align the filtered reads to the reference genome [11].

### 2.5. Analysis of Differential Expression

Differentially expressed genes (DEGs) were analyzed using slightly altered versions of accepted techniques. As a preliminary indicator of gene expression, read count values for each gene were compared using HTSeq [12]. To normalize gene expression, FPKM (fragments per kilobase of transcript per million mapped reads) was used. DESeq was then utilized to identify DEGs based on a fold change (FC) of greater than 2 (or less than 0.5) and a significant *p*-value below 0.05. All DEGs were subjected to a bi-directional clustering analysis using Pheatmap. Full linkage clustering and Euclidean distance calculations were used to generate a heatmap that depicted the expression patterns of genes within and across groups.

### 2.6. Analyses of GO and KEGG Enrichments

Gene Ontology (GO) concepts were assigned to all genes, and the differential enrichment of genes was determined for each term [13]. The GO enrichment analysis of differentially expressed genes (DEGs) was performed using the topGO program. The hypergeometric distribution was employed to calculate *p*-values, with a significant enrichment threshold set at *p* < 0.05. GO keywords associated with significantly differentially enriched genes were identified and categorized to elucidate their primary biological roles. Furthermore, ClusterProfiler software V3 was used to examine the enrichment of DEGs in KEGG pathways, employing the same criterion of *p* < 0.05 to indicate significant enrichment [14].

### 2.7. Analysis of SNP Sites

In delving into the analysis of single nucleotide polymorphism (SNP) sites, our study employed the Varscan 2 software as the analytical tool of choice, applying stringent criteria for the identification of SNP locations to ensure the precision and reliability of the outcomes. Sites were required to have a minimum coverage of 8 reads, with at least 2 reads supporting the mutation. Furthermore, a *p*-value threshold of <0.01 was applied for the SNP locus, alongside a base quality score (Q) cutoff exceeding 20. This approach ensured the reliability and accuracy of identified SNPs, facilitating insights into genetic variation and its functional significance [15].

### 2.8. Analysis of Differential Exon Usage

Using the DEXSeq 2 software, an investigation of differential exon use was carried out while strictly adhering to previously defined techniques [16]. This computational tool facilitated the examination of RNA-seq data for variations in exon inclusion frequencies across different conditions. Through this approach, significant alterations in exon usage were elucidated, potentially enhancing the comprehension of gene regulatory mechanisms and illuminating the functional significance of alternative splicing within biological pathways.

### 2.9. Analysis of Interactions in Protein Networks

The utilization of the STRING database facilitated an intricate analysis of the protein network, thereby elucidating potential protein–protein interaction (PPIs) [17]. We were able to fully comprehend the underlying biological pathways and molecular mechanisms by using this ingenious technology to clarify the complex interactions and connectivity between the genes of interest.

### 2.10. Analysis of GSEA Enrichments

By ranking genes according to differences in their expression between two sample groups, Gene Set Enrichment Analysis (GSEA) does away with the need for a rigid threshold for differential gene classification [18]. The subsequent step involves employing statistical methods to ascertain whether enriched gene sets are present at the top or bottom of the sorted list. Calculating the enrichment score, assessing its significance, and performing multiple hypothesis testing are the core processes in GSEA. For the species under investigation, GSEAs were conducted on the Gene Ontology (GO), Kyoto Encyclopedia of Genes and Genomes (KEGG), Reactome, Disease Ontology (DO), and DisGeNET databases.

### 2.11. Validation of Gene Expression by RT-qPCR

To validate the gene expression profile, four representative genes—Lgfb5, Ler2, Vgll3, and Ahr—were selected. As an internal reference standard, GAPDH was employed. Following the manufacturer’s instructions, three technical replicates of the RT-qPCR analysis were carried out using the qPCR SYBR Green Master Mix (Vazyme, Nanjing, China) and the LightCycler 480^®^ Real-Time PCR System (Roche, Rotkreuz, Switzerland). The 2^−ΔΔCt^ method was employed to interpret the RT-qPCR results and quantify the relative expression levels of the target genes. GraphPad Prism (v8.0) was used for statistical analysis, which included Welch’s correction and a two-tailed Student’s *t*-test. Three separate studies’ means ± standard deviations are used to display the findings.

## 3. Results

### 3.1. De Novo Transcriptome Assembly via Sequencing

**Figure 1:**
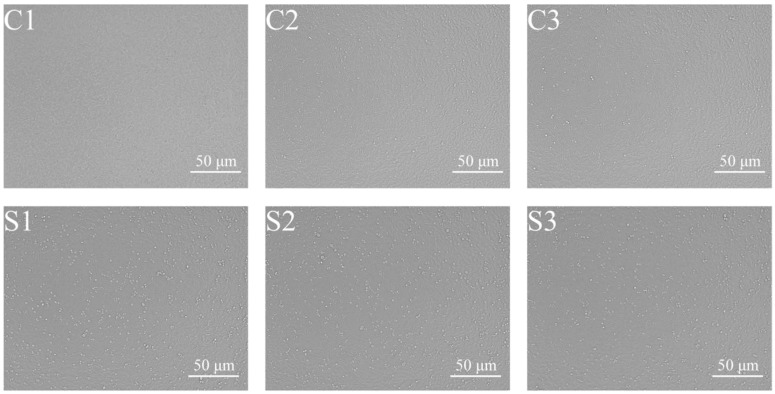
Monolayers of cells with and without FMDV infection at 12 hpi. C1, C2, and C3 are non-infected controls; S1, S2, and S3 are FMDV-infected groups.

At 12 h post-infection (hpi), BHK-21 cell monolayers displayed a minimal cytopathic effect (CPE) (Figure 1). Total RNA was extracted from both FMDV-infected and non-infected cell groups to create premium cDNA libraries. These libraries were sequenced to generate image files, which were then converted into raw data for statistical analysis. The raw data contained numerous adapters and low-quality reads. To enhance data quality, Cutadapt (v1.15) software was employed, considering base quality, base composition, and average read quality as the primary factors (Table 1). In our RNA sequencing analysis, all samples successfully obtained high-quality sequencing data, with read counts ranging from 468 million to 596 million and corresponding sequence data volumes of 7.03 to 8.94 gigabytes, with an error rate of only 0.01%. The read quality of each sample was very high, with Q20 and Q30 rates reaching 99.15–99.23% and 97.49–97.75%, respectively. Samples from both the S group and C group exhibited similar high data quality, confirming the consistency and accuracy of the sequencing process.

### 3.2. Transcriptomic Mapping

Filtered reads were aligned to the reference genome of Mesocricetus auratus using HISAT2. Table 2 presents the results of the RNA-seq mapping. The distribution of reads mapped to the genome, including coding sequences, introns, intergenic spacers, and UTRs, underwent detailed statistical analysis. Table 3 offers details on the mapping outcomes. Overall, a high-quality RNA-seq dataset that meets the criteria for further bioinformatics analysis was obtained.

### 3.3. Profile of Gene Expression

**Figure 2:**
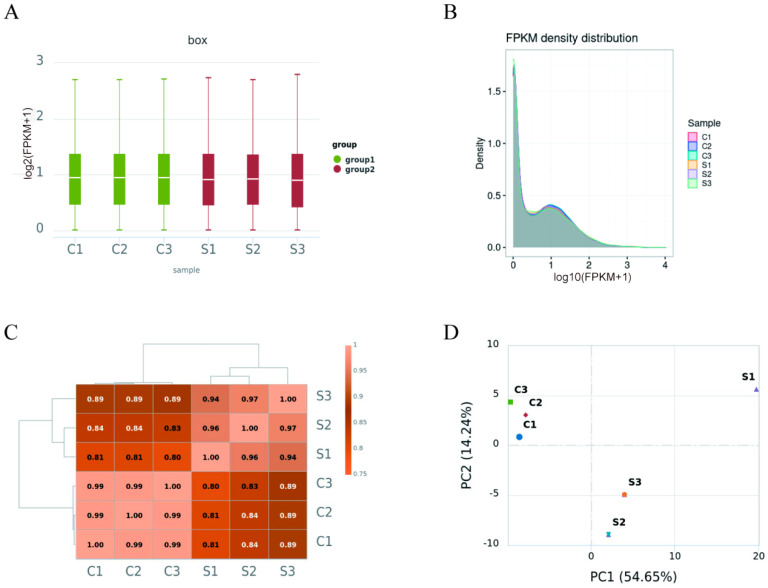
Gene expression profiles across all groupings. FPKM distributions in two groups in a boxplot (**A**). Density distributions of FPKM in two groups (**B**). The computation of Pearson’s correlation coefficients (**C**) for the correlation analysis of gene expression. Study of principal components for two groups (**D**). Primary component one is PC1, while primary component two is PC2.

FPKM (fragments per kilobase of transcript per million mapped reads) is a simple technique that considers both gene length and the total number of mapped reads in order to normalize read count data. FPKM-normalized expression levels were assigned different intervals. Two visual representations of the FPKM value density distribution are provided in Figure 2A,B. Pearson’s correlation coefficients were used to analyze gene expression correlations across the six groups (Figure 2C), where a correlation coefficient close to 1.0 indicates greater similarity in expression patterns. Additionally, as shown in Figure 2D, high-dimensional datasets can be projected onto two or three dimensions using principal component analysis (PCA), with increased similarity between groupings indicated by greater distance between the spots.

### 3.4. Analysis of Differential Expression

**Figure 3:**
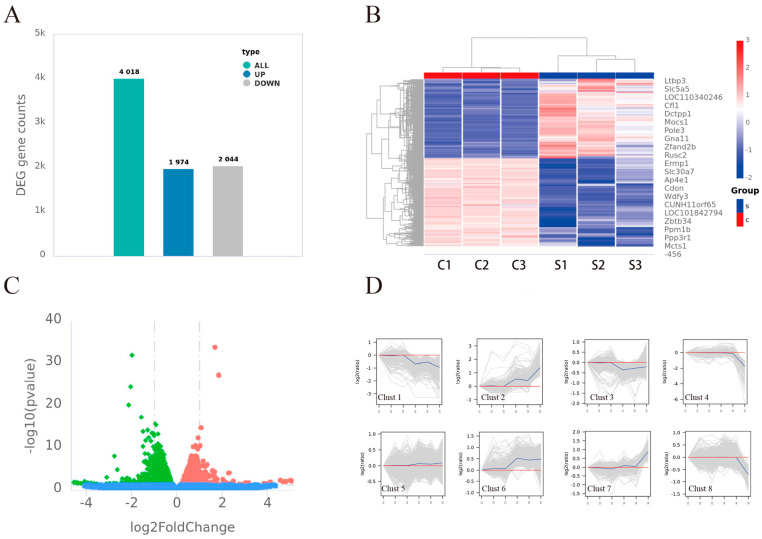
DEG profile and analysis. Upregulated and downregulated genes make up the bar graph of total DEGs (**A**). Plot of the *p*-value against FC for every gene found, eliminating outliers (**B**). |log2FC| > is the threshold value selected. *p*-value <0.05 and 1. Heatmap of all DEGs based on bi-directional clustering technique (**C**). Analysis of DEG expression patterns using clustering (**D**). Patterns of expression are indicated by gray lines. The average value in each cluster is shown by each blue line. The green dots now represent genes with decreased expression, while the red dots represent genes with increased expression.

Differentially expressed genes (DEGs) were found using DESeq (v1.30.0) based on the screening criterion of |log2FC| > 1 and a significant *p*-value < 0.05. There were 4018 DEGs found in all, with 2044 downregulated and 1974 upregulated genes. A total of 4018 DEGs were identified, comprising 2044 downregulated and 1974 upregulated genes (Figure 3A). A heatmap and a volcano plot were used to graphically represent the distribution and magnitude of differential expression. The volcano plot (Figure 3B), generated using GraphPad Prism 9 software, depicts the relationship between *p*-values and fold changes (FC) for all identified genes, with red, green, and blue circles representing upregulated, downregulated, and stably expressed genes, respectively. The heatmap (Figure 3C), which graphically depicts the hierarchical clustering of all 4018 DEGs across the six groups, was created using the Pheatmap (1.0.8) package in R and a bi-directional clustering algorithm. The color intensity of the heatmap indicates the degree of expression difference, whereas the red and blue labels denote upregulated and downregulated DEGs, respectively. All 4018 DEGs were categorized into eight clusters based on their expression patterns (Figure 3D), with each blue line representing the average expression value for its cluster and gray lines showing individual expression profiles.

### 3.5. GO Enrichment Analysis

**Figure 4:**
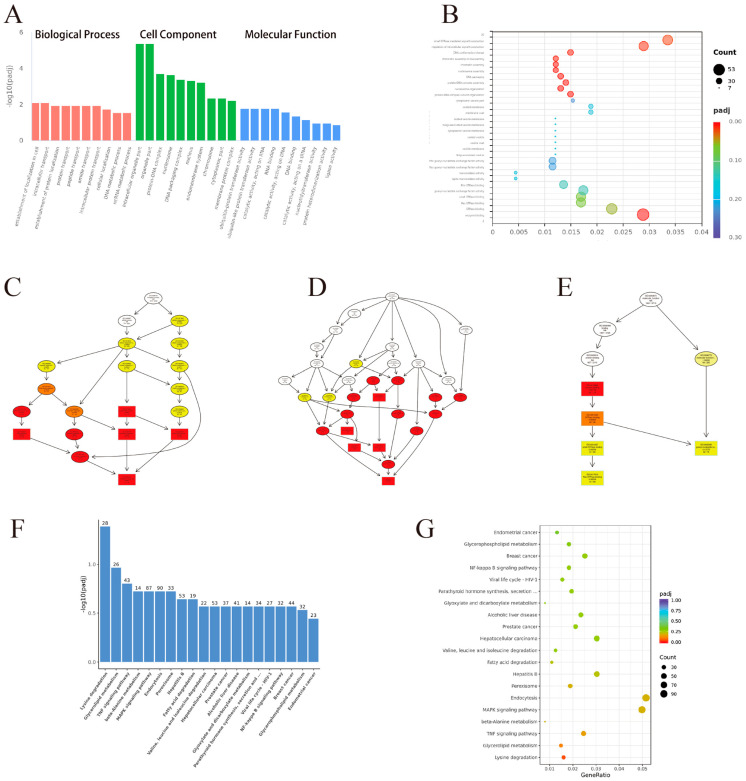
DEGs’ KEGG and GO enrichment analysis. (**A**) The ten most important GO phrases in each of the three categories. The top 20 statistically significant GO keywords are shown in a bubble plot (**B**). Acyclic graphs that show the top ten statistically significant GO categories were directed (**C**–**E**). Each GO term’s color intensity corresponds to its statistical significance; higher importance is indicated by darker hues. (**F**) The 20 KEGG pathways that are statistically significant. (**G**) The top 20 statistically significant KEGG pathways are shown in a bubble plot.

The topGO tool was used to conduct a Gene Ontology (GO) enrichment analysis on the differentially expressed genes (DEGs). This study identified GO keywords with significant differential enrichment. There were 417, 114, and 269 entries in the categories for Biological Process (BP), Cellular Component (CC), and Molecular Function (MF), respectively. The top 10 statistically significant GO keywords for each category are presented in Figure 4A. The false discovery rate (FDR) indicates the degree of GO enrichment, ranging from 0 to 1, with lower FDR values indicating higher significance. A bubble plot in Figure 4B displays the 20 lowest FDR GO keywords. Directed acyclic graphs (Figure 4C–E) depict the hierarchical structure of each GO category, with parent terms encompassing broader functional categories than their child terms. Rectangles represent GO terms with the 10 lowest FDRs, while ellipses indicate the remaining terms. The color intensity of each GO term corresponds to its statistical significance, with deeper colors indicating greater relevance.

### 3.6. KEGG Enrichment Analysis

The KEGG pathway enrichment analysis was used to determine the pathways linked to the differentially expressed genes (DEGs). Figure 4F shows the top 30 statistically significant KEGG pathways (*p*-value < 0.05). The enrichment analysis results were assessed based on the enrichment factor, false discovery rate (FDR), and the number of DEGs linked to each pathway. Lower FDR values indicate higher significance in enrichment, while higher enrichment factors suggest a greater degree of enrichment. The bubble plot in Figure 4G illustrates the 20 KEGG pathways with the lowest FDRs.

### 3.7. Additional RNA-seq Data Analysis

**Figure 5:**
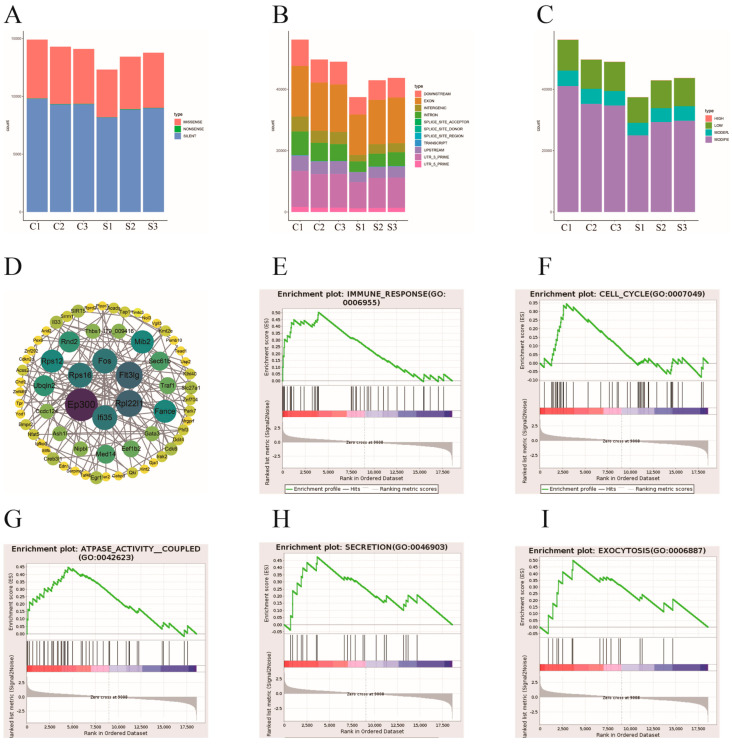
In-depth analysis of RNA-seq data. The variation sites according to functional classification (**A**). The variation sites based on regional classification (**B**). The variation sites categorized by their impact (**C**). PPI network (**D**). Significant enrichment in activities including “secretion”, “exocytosis”, “immune response”, “ATPase activity coupled”, and “cell cycle” was found by Gene Set Enrichment Analysis (GSEA) (**E–I**).

Varscan was employed to assess SNP locations, and the functional categorization of each sample’s variation sites is illustrated in Figure 5A. Figure 5B depicts the variation sites geographically, while Figure 5C shows them by their impact. Furthermore, differentially expressed genes (DEGs) in the STRING database were carefully examined to find putative PPIs with a score greater than 0.95 in order to make the process of building a protein–protein interaction (PPI) network easier (Figure 5D). Significant enrichment was seen in processes including “secretion”, “exocytosis”, “immune response”, “ATPase activity coupled”, and “cell cycle” according to Gene Set Enrichment Analysis (GSEA) (Figure 5E–I).

### 3.8. RT-qPCR Validation of Gene Expression

**Figure 6:**
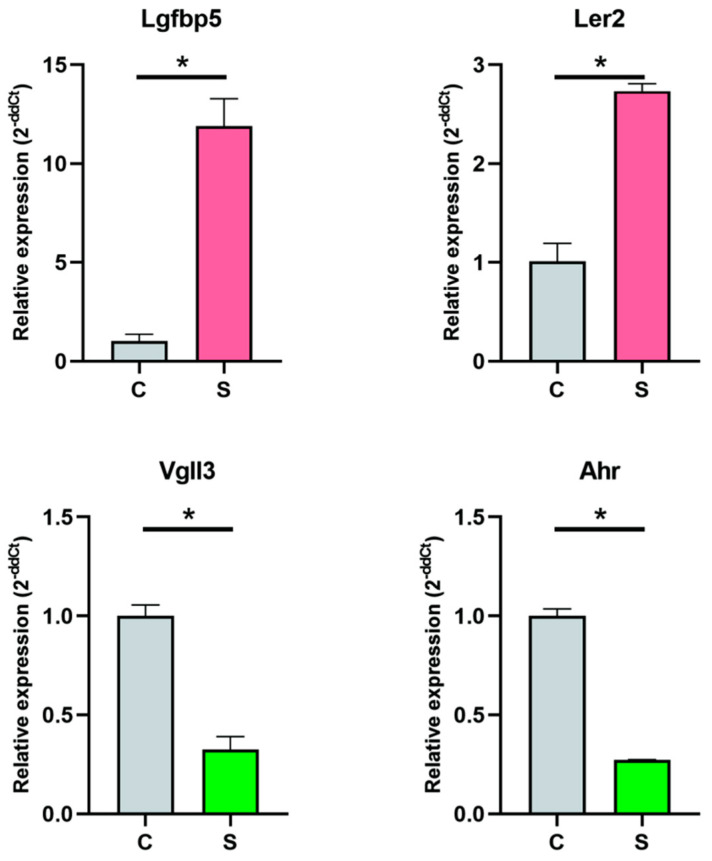
Verification of gene expression using RT-qPCR. The 2^−ΔΔCt^ technique was used for relative quantification, and the GAPDH gene was used as an internal reference to ensure normalization. Three separate experiments’ means ± standard deviations are used to display the data. A two-tailed Student’s *t*-test with Welch’s correction was used to assess statistical significance; * *p* < 0.05 denotes significant differences. Grey represents the control samples, red indicates samples with increased expression, and green indicates samples with decreased expression.

Two upregulated and two downregulated differentially expressed genes (DEGs) were chosen in order to validate the gene expression patterns using RT-qPCR. These were designated as Lgfb5 and Ler2 for the upregulated genes and Vgll3 and Ahr for the downregulated ones. The DEGs’ expression patterns matched the results of the RNA-seq investigation according to the RT-qPCR analysis (Figure 6).

## 4. Discussion

The Picornaviridae family, in the family of plus-strand RNA viruses, is well characterized, with FMDV as a representative picornavirus [19]. The positive-sense single-stranded mRNA that makes up the FMDV genome has a 3′ poly(A) tail but no 5′ cap structure. The picornaviral genome enters the host cell and is released into the cytoplasm, where it serves as a template for the production of polyproteins or an antigenome [20]. This process can significantly impact the host’s physiological processes. Transcriptome sequencing provides a broad viewpoint on the dynamics of gene expression, enabling the identification of transcripts that have not been previously identified, the disclosure of alternative splicing occurrences, and an understanding of the complexities of gene regulatory mechanisms [21,22]. In the field of virology, this technology is invaluable, since it allows for the accurate mapping of viral transcripts and a thorough investigation of the viral–host interaction [23]. It advances our knowledge of the host immune system, the progression of the illness, and viral latency, creating new opportunities for the identification of therapeutic targets and the creation of innovative antiviral tactics. As a result, transcriptome sequencing represents a major advancement in our knowledge of and ability to treat viral infections [24,25]. Our investigation into the transcriptome changes in FMDV-infected cells was motivated by the understanding that FMDV infection can profoundly affect the host transcriptome despite the absence of a traditional viral transcriptome.

Twelve hours after being inoculated with FMDV at a MOI of 0.1, three cell monolayers showed negligible cytopathic effects (CPEs) (Figure 1). Our research indicates that at this stage, FMDV infection leads to significant alterations in proteome and metabolomic profiles. A high correlation coefficient, ranging from 0.8 to 1.0, is indicative of a strong correlation between the two groups, which is a critical metric for assessing sample validity and the reliability of the experiment. The correlation analysis conducted in our study revealed a poor correlation between groups but a strong intra-group correlation (Figure 2C), suggesting the validity of the RNA-seq data.

To further explore differential expression, RNA-seq data were subjected to additional analysis. The differential expression analysis identified 1974 upregulated and 2044 downregulated genes, which were subsequently analyzed to confirm the expression patterns by RT-qPCR. Four (Lgfb5, Ler2, Vgll3, Ahr) of these DEGs were selected for RT-qPCR validation to preliminarily confirm their expression patterns. The RT-qPCR results concurred with the RNA-seq data (Figure 6), indicating the validity of the RNA-seq findings. KEGG enrichment analysis highlighted the significant enrichment of DEGs in immunity-related pathways, such as the MAPK, NF-kappa B, and TNF signaling pathways (Figure 4G). In virology, the MAPK pathway is instrumental in regulating viral replication and pathogenesis, making it a significant target for antiviral strategies and therapeutic interventions [26,27]. NF-kappa B activation is frequently manipulated by viruses to create a favorable intracellular environment for their replication [28,29]. Viruses can both activate and inhibit NF-kappa B signaling to evade the host immune system, manipulate apoptosis, and enhance viral gene expression [30]. Understanding the intricate interplay between viral pathogens and the NF-kappa B pathway is essential for developing novel antiviral strategies that target the disruption of these viral exploitation mechanisms [31]. In virology, TNF signaling is a double-edged sword; while it can inhibit viral replication, certain viruses have evolved mechanisms to exploit these pathways for their benefit [32]. They may dysregulate TNF-induced apoptosis to favor viral persistence or use the inflammatory response to create a suitable environment for spread [33]. Thus, targeting TNF signaling is a promising strategy for developing antiviral therapies that disrupt the viral manipulation of host responses [34]. Additionally, GO enrichment analysis revealed numerous statistically significant terms related to material movement, including intracellular transport, protein transport, peptide transport, and amide transport (Figure 4A).

A comprehensive analysis of the RNA-seq data was conducted, focusing on single nucleotide polymorphisms (SNPs), protein–protein interactions (PPIs), and Gene Set Enrichment Analysis (GSEA). There was no discernible variation in the frequency of SNP events between the control group C and the FMDV-infected group S, according to the analysis of SNP occurrences (Figure 5A–C), indicating that FMDV infection does not cause SNP events in the host’s genome. Employing the STRING database, putative protein–protein interactions (PPIs) in cells infected with FMDV were delineated. Figure 5D illustrates that Fos, Flt3lg, Rpl22l1, Ifi35, Ep300, and Rps16 were identified as important hub proteins due to their high connection degrees in the PPI network. GSEA results (Figure 5E–I) revealed a positive association between FMDV and several biological processes, such as immunological response, cell cycle control, ATPase activity-related processes, and secretion and exocytosis signaling pathways.

Over the past two decades, FMDV has been detected in numerous countries and regions worldwide [35]. The virus’s evolutionary trajectory has been heavily influenced by natural selection, particularly in codon usage bias. Employing multi-omics approaches offers a comprehensive method to enhance our understanding of viral biology, especially regarding the virus–host interactions [36]. The aim of this study was to compare the transcriptomic profiles of FMDV-infected cells with those of uninfected cells. Our findings indicate that the majority of DEGs were upregulated, suggesting that FMDV infection may enhance host cell transcription initiation. Protein–protein interaction network analysis identified six key hub proteins (Fos, Flt3lg, Rpl22l1, Ifi35, Ep300, and Rps16). Several statistically significant terms associated with material mobility were found by GO enrichment analysis. Moreover, KEGG analysis revealed that FMDV significantly impacts immunity-related cellular pathways, although the underlying mechanisms of these effects remain elusive.

Previous studies on FMDV transcriptomics have focused on host gene expression during persistent infection, changes in porcine PBMCs post-infection, and apoptosis in bovine tonsil tissue. Despite BHK-21 cells’ common use for FMDV culture, their transcriptional changes remain underexplored. Our data reveal distinct gene expression patterns in infected versus control BHK-21 cells. Li J’s team found acute and persistent FMDV infections in BHK-21 cells altered thousands of genes, mainly in metabolic and ribosomal pathways [37]. Zhao FR’s study on pig PBMCs post-FMDV infection highlighted immune signaling pathways but noted that FMDV does not replicate in PBMCs [38]. Han L’s work on FMDV-resistant BHK-21 cells showed upregulated genes linked to metabolism and viral response [39]. Eschbaumer M suggested FMDV persistence in cattle involves apoptosis and regulatory T-cell activity [40]. Saravanan S’s analysis of infected cattle tonsils revealed metabolic and innate immune responses, especially in indigenous breeds [41]. These findings deepen our understanding of FMDV’s host impact and suggest targets for treatments and diagnostics, crucial for disease control and economic relief.

## Figures and Tables

**Table 1:** RNA-seq statistics for each of the six categories.

Sample	Raw_Reads	Raw_Bases	Error_Rate	Q20	Q30
C1	59608728	8.94 G	0.01	99.23	97.75
C2	48456016	7.27 G	0.01	99.15	97.49
C3	46872584	7.03 G	0.01	99.18	97.57
S1	51425968	7.71 G	0.01	99.19	97.56
S2	50985892	7.65 G	0.01	99.19	97.58
S3	51126662	7.67 G	0.01	99.2	97.6

**Table 2:** Data from RNA-seq mapping statistics.

Sample	Total_Reads	Total_Map	Unique_Map	Multi_Map
C1	57,923,034	53,335,422 (92.08%)	51,050,372 (88.13%)	2,285,050 (3.94%)
C2	47,517,542	44,024,204 (92.65%)	42,258,755 (88.93%)	1,765,449 (3.72%)
C3	45,965,232	42,241,759 (91.9%)	40,624,971 (88.38%)	1,616,788 (3.52%)
S1	50,429,350	39,210,770 (77.75%)	37,623,031 (74.61%)	1,587,739 (3.15%)
S2	49,797,192	40,176,416 (80.68%)	38,469,646 (77.25%)	1,706,770 (3.43%)
S3	49,814,424	40,082,756 (80.46%)	38,523,751 (77.33%)	1,559,005 (3.13%)

**Table 3:** Read-mapped regions’ distribution.

Sample	Exon	Intron	Intergenic
C1	7,285,406,051 (91.1852%)	306,126,883 (3.8315%)	398,150,357 (4.9833%)
C2	6,033,236,422 (91.4872%)	241,780,946 (3.6663%)	319,609,062 (4.8465%)
C3	5,823,037,349 (92.0338%)	229,090,137 (3.6208%)	274,940,524 (4.3455%)
S1	5,532,463,177 (94.1956%)	125,114,966 (2.1302%)	215,801,808 (3.6742%)
S2	5,586,065,790 (92.8252%)	173,362,395 (2.8808%)	258,400,739 (4.2939%)
S3	5,575,275,393 (92.8603%)	177,995,772 (2.9646%)	250,670,374 (4.1751%)

## Data Availability

FMDV-infected cells and uninfected cell sequencing data have been deposited in the NCBI database under project PRJNA1177641 with accession numbers SAMN44445761, SAMN44445762, SAMN44445763, SAMN44445764, SAMN44445765, and SAMN44445766.
